# Toward the Use of Methyl-Coenzyme M Reductase for Methane Bioconversion Applications

**DOI:** 10.1021/acs.accounts.4c00413

**Published:** 2024-08-27

**Authors:** Thuc-Anh Dinh, Kylie D. Allen

**Affiliations:** Department of Biochemistry, Virginia Tech, Blacksburg, Virginia 24061, United States

## Abstract

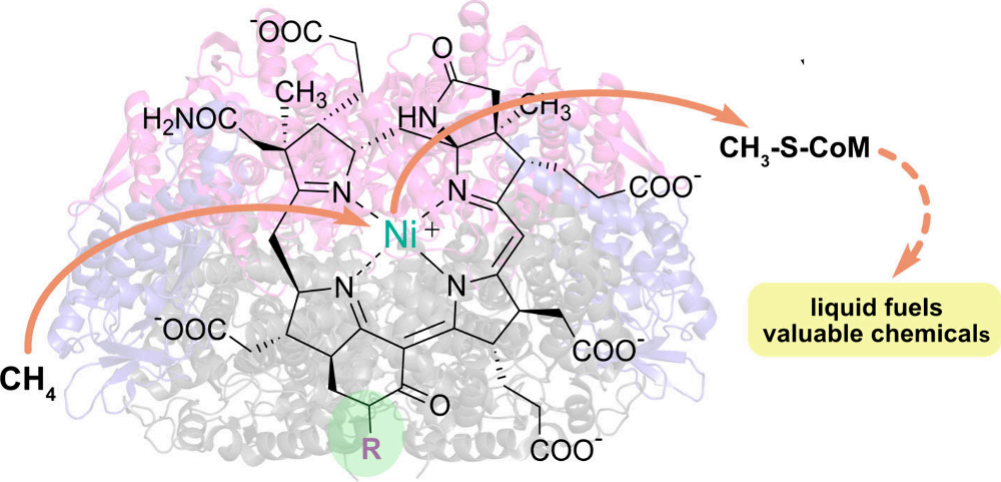

As the main component of natural gas and renewable biogas, methane is an abundant, affordable fuel. Thus, there is interest in converting these methane reserves into liquid fuels and commodity chemicals, which would contribute toward mitigating climate change, as well as provide potentially sustainable routes to chemical production. Unfortunately, specific activation of methane for conversion into other molecules is a difficult process due to the unreactive nature of methane C–H bonds. The use of methane activating enzymes, such as methyl-coenzyme M reductase (MCR), may offer a solution. MCR catalyzes the methane-forming step of methanogenesis in methanogenic archaea (methanogens), as well as the initial methane oxidation step during the anaerobic oxidation of methane (AOM) in anaerobic methanotrophic archaea (ANME). In this Account, we highlight our contributions toward understanding MCR catalysis and structure, focusing on features that may tune the catalytic activity. Additionally, we discuss some key considerations for biomanufacturing approaches to MCR-based production of useful compounds.

MCR is a complex enzyme consisting of a dimer of heterotrimers with several post-translational modifications, as well as the nickel-hydrocorphin prosthetic group, known as coenzyme F_430_. Since MCR is difficult to study *in vitro*, little information is available regarding which MCRs have ideal catalytic properties. To investigate the role of the MCR active site electronic environment in promoting methane synthesis, we performed electric field calculations based on molecular dynamics simulations with a MCR from *Methanosarcina acetivorans* and an ANME-1 MCR. Interestingly, the ANME-1 MCR active site better optimizes the electric field with methane formation substrates, indicating that it may have enhanced catalytic efficiency. Our lab has also worked toward understanding the structures and functions of modified F_430_ coenzymes, some of which we have discovered in methanogens. We found that methanogens produce modified F_430_s under specific growth conditions, and we hypothesize that these modifications serve to fine-tune the activity of MCR.

Due to the complexity of MCR, a methanogen host is likely the best near-term option for biomanufacturing platforms using methane as a C1 feedstock. *M. acetivorans* has well-established genetic tools and has already been used in pilot methane oxidation studies. To make methane oxidation energetically favorable, extracellular electron acceptors are employed. This electron transfer can be facilitated by carbon-based materials. Interestingly, our analyses of AOM enrichment cultures and pure methanogen cultures revealed the biogenic production of an amorphous carbon material with similar characteristics to activated carbon, thus highlighting the potential use of such materials as conductive elements to enhance extracellular electron transfer.

In summary, the possibilities for sustainable MCR-based methane conversions are exciting, but there are still some challenges to tackle toward understanding and utilizing this complex enzyme in efficient methane oxidation biomanufacturing processes. Additionally, further work is necessary to optimize bioengineered MCR-containing host organisms to produce large quantities of desired chemicals.

## Key References

- PolêtoM. D.; AllenK. D.; LemkulJ. A.Structural Dynamics of the Methyl-Coenzyme M Reductase Active Site Are Influenced by Coenzyme F_430_ Modifications. Biochemistry2024, 63( (14), ), 1783–179438914925 10.1021/acs.biochem.4c00168PMC11256747.^1^ Molecular dynamics simulations of *M. acetivorans* and ANME-1 MCRs with canonical vs modified versions of coenzyme F_430_. The modifications have substantial impacts on active site structure and dynamics. Further, electric field calculations suggest differences in catalytic efficiencies.
- AllenK. D.; WegenerG.; WhiteR. H.Discovery of Multiple Modified F_430_ Coenzymes in Methanogens and Anaerobic Methanotrophic Archaea Suggests Possible New Roles for F_430_ in Nature. Appl. Environ. Microbiol.2014, 80, 6403–641225107965 10.1128/AEM.02202-14PMC4178637.^2^ This is the first report of modified F_430_s existing in several methanogens. We proposed the structure of the modifications based on mass spectra, UV–vis spectra, and some chemical characterization.
- AllenK. D.; WegenerG.; Matthew SublettD.Jr.; BodnarR. J.; FengX.; WendtJ.; WhiteR. H.Biogenic Formation of Amorphous Carbon by Anaerobic Methanotrophs and Select Methanogens. Sci. Adv.2021, 7, eabg973934705502 10.1126/sciadv.abg9739PMC8550235.^3^ Here, we describe the identification and characterization of an amorphous black carbon material produced in two different AOM consortia. Additionally, pure cultures of some methanogens produce a similar carbon material.

## Introduction

Methane is the major component of natural gas, which is an important energy source worldwide. In the last few decades, improved drilling and extraction technologies have revealed an abundance of natural gas reserves and led to low natural gas prices compared to many other fuels. Additionally, microbially produced methane in biogas generated during anaerobic digestion is a sustainable and renewable energy source. On the other hand, methane is a potent greenhouse gas (GHG) that currently comprises ∼21% of global GHG emissions and has a warming potential at least 25 times greater than carbon dioxide.^4^ This has contributed to interests in converting methane into more convenient liquid fuels and other valuable chemicals.^5^ The abundance and low cost of methane coupled with its high degree of reduction makes it an appealing C1 substrate for chemical manufacturing. Furthermore, metabolic flux balance analyses indicate that methane is the lowest cost feedstock for microbial biomanufacturing compared to methanol, carbon monoxide, and acetate.^6^ However, methane C–H bonds are the strongest (+439 kJ/mol, 105 kcal/mol) of the alkanes, making specific and selective activation of methane an especially difficult task. The current gas-to-liquids (GTL) technology for natural gas conversion to longer hydrocarbons involves steam reforming processes to generate syngas (H_2_ and CO) followed by the well-established Fischer–Tropsch synthesis.^7^ This GTL technology requires expensive large-scale industrial facilities, generates significant GHG emissions, and has low carbon conversion efficiency.^5,8^ A promising alternative to chemical methane activation is to utilize biological/enzymatic systems in biological GTL (Bio-GTL) processes, which would be less technologically complex, thus supporting small-scale facilities while improving carbon conversion efficiency.^5,8,9^

There are only a few enzymes known to catalyze methane oxidation: particulate methane monooxygenase (pMMO), soluble methane monooxygenase (sMMO), methyl-coenzyme M reductase (MCR), and cytochrome P450s.^9,10^ Much work and interest has focused on MMOs and their methanotrophic bacterial host organisms for potential use in methane bioconversion applications, with many groundbreaking advances.^11^ As monooxygenases, MMOs and P450s utilize dioxygen as a substrate to activate methane, generating methanol and water. These enzymes require two electrons—derived from NAD(P)H *in vivo*—for each methane oxidized. Notably, these aerobic pathways are overall less energy and carbon efficient compared to the anaerobic pathway initiated by MCR.^5,9^

Here, we focus on anaerobic methane metabolism orchestrated by MCR. This enzyme catalyzes the methane-forming step of methanogenesis in methanogenic archaea as well as the initial methane oxidation step during the anaerobic oxidation of methane (AOM) in anaerobic methanotrophic archaea (ANME).^12^ Additionally, MCR homologs—alkyl-coenzyme M reductases (ACRs)—catalyze the initial C–H activation step in the oxidation of other alkanes outside of methane.^13,14^ Thus, MCR/ACR-based production of fuels and other commodity chemicals has great potential. In this Account, we summarize the current state of the field and highlight our contributions toward understanding MCR catalysis and structure, especially focused on features that may enhance the catalytic activity or promote the methane oxidation reaction. Additionally, we discuss considerations for biomanufacturing approaches to MCR-based production of useful compounds, including the use of carbon-based materials to facilitate AOM.

## Overview of Anaerobic Methane Metabolism

**Figure 1:**
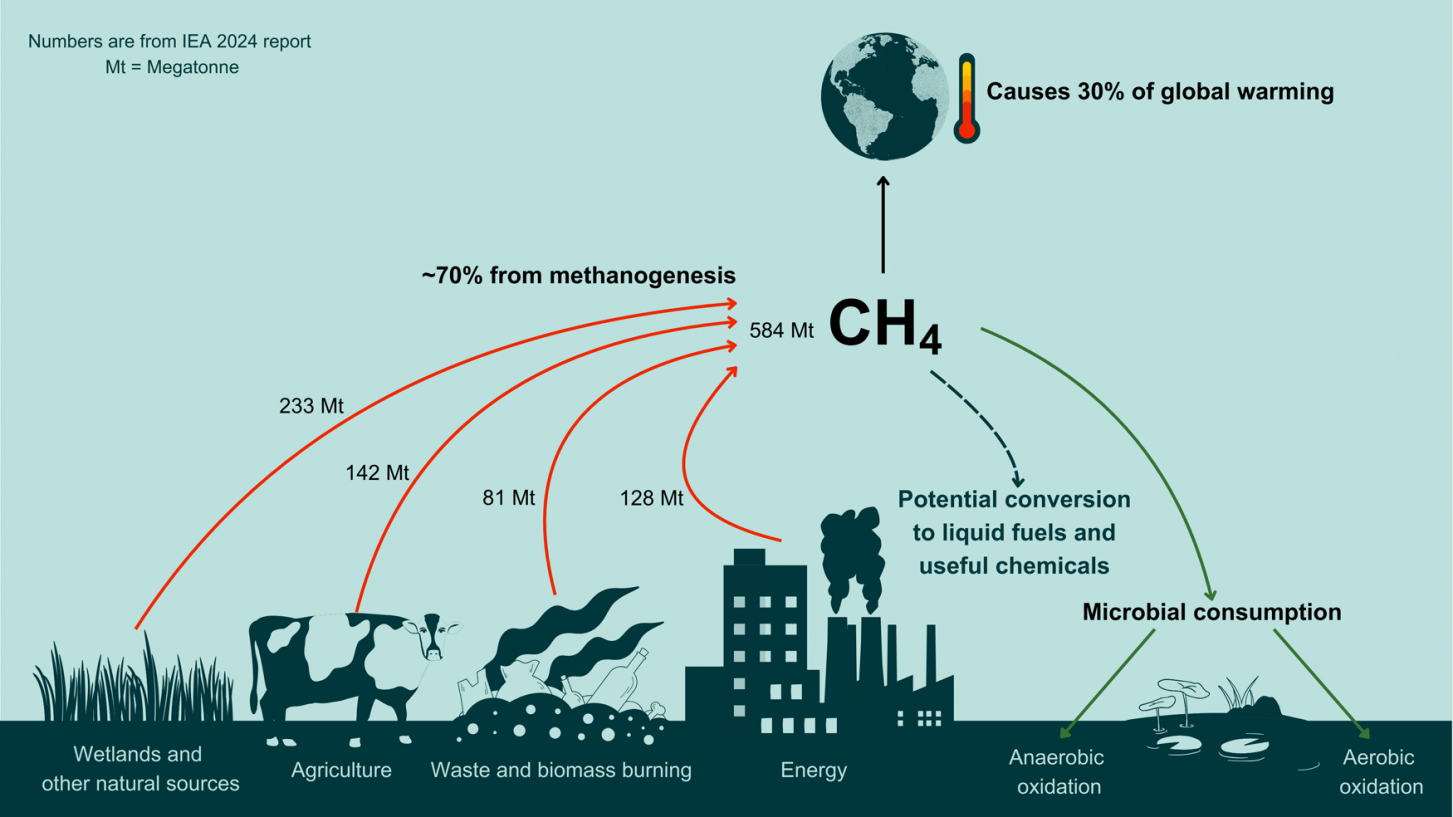
Overview of the global methane budget. Information from the International Energy Agency (IEA) 2024 report.^19^

MCR was first discovered and has been most well-characterized in methanogenic archaea (“methanogens”), where it operates in the thermodynamically favorable methane formation direction to catalyze the ultimate reaction of methanogenesis, the essential energy metabolism of these organisms. Methanogens are ubiquitous in anaerobic environments—from wetlands, landfills, and gut microbiomes to marine sediments and hydrothermal vents.^15^ Methanogenesis is a form of anaerobic respiration that involves the conversion of a variety of carbon compounds—including CO_2_, methyl compounds, and acetate—to produce methane as the end product. This process is responsible for at least 70% of global methane emissions (Figure 1).^19^

ANME are phylogenetically closely related to methanogens and utilize a reverse methanogenesis pathway to carry out AOM in methane-rich environments. ANME are divided into three taxonomic groups: ANME-1 (*Ca*. Methanophagales), ANME-2 (subclusters a, b, c, and d), and ANME-3.^13,17^ Most ANME exist in consortia with sulfate-reducing bacteria (SRB) that allow AOM to be coupled with sulfate reduction.^17,18^ The only known ANME that do not couple AOM with syntrophic sulfate reduction are the *Ca*. Methanoperedenaceae (ANME-2d), which occupy freshwater environments and utilize nitrate or oxidized metals as electron acceptors.^17,16^ In contrast to well-studied methanogens, experimental investigation of ANME physiology, genetics, and biochemistry is hindered by their extended lag phases, slow growth rates, low growth yields, and low cell densities, as well as their dependence on bacterial partners.

## MCR Reactions, Mechanisms, and Kinetics

**Figure 2:**
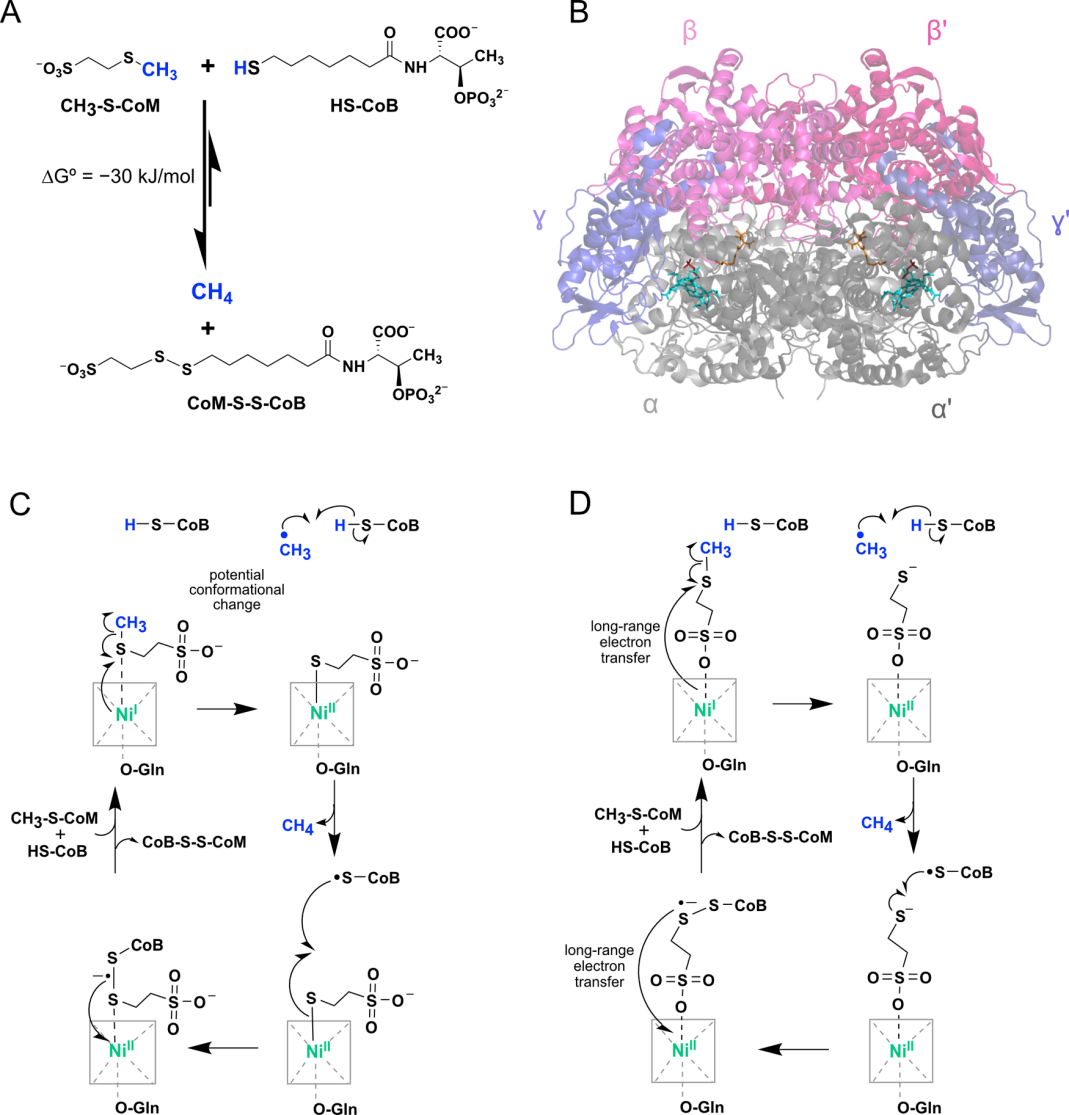
MCR reaction, structure, and mechanism. (A) The methane formation and reverse methane oxidation reactions catalyzed by MCR. (B) Overall structure of *M. marburgensis* MCR I (PDB 5A0Y) with F_430_ (teal), HS-CoM (dark red), and HS-CoB (orange). (C and D) Proposed mechanisms for MCR catalysis, based on Wongnate et al. (C)^23^ and Patwardhan et al. (D).^26^

Methanogenic MCR has been extensively studied in the methane formation direction, where it catalyzes the conversion of methyl-coenzyme M (CH_3_-S-CoM) and coenzyme B (HS-CoB) to methane and the CoM-S-S-CoB heterodisulfide (Figure 2A).^12^ The enzyme is a dimer of heterotrimers consisting of 2α, 2β, and 2γ subunits with two active sites harboring the nickel-tetrahydrocorphin coenzyme F_430_, which is the key catalytic machinery (Figure 2B).^20^ In both methanogens and ANME, MCR is highly expressed, which makes the native protein relatively easy to purify assuming the availability of large amounts of the relevant biomass.

The current state of knowledge regarding MCR catalysis comes from work by several research groups primarily on the natively purified MCR from *Methanothermobacter marburgensis*. This hydrogenotrophic, thermophilic methanogen grows to very high cell densities and effective protocols are in place for the isolation of active (F_430_ with Ni(I)) or activatable (Ni(III)) forms of MCR from this organism.^21^ The Ni(I) form is highly susceptible to oxidative inactivation (even when isolated under “strictly” anaerobic conditions) to produce Ni(II) (“silent” due to being EPR silent), which cannot be reduced to the active form *in vitro* with chemical reductants. The inactive Ni(II) state is often the predominant form resulting upon cell lysis in most organisms and conditions, leading to the overall scarcity of MCR enzymology in the literature. It is important to note here that although genetic tools are well-developed in a handful of model methanogens, there are no reports of activity experiments with recombinant MCRs, likely due to the inability to obtain active forms of these enzymes. Thus, traditional mutagenesis experiments to investigate the importance of specific amino acid residues are not yet feasible for MCR.

The working mechanism for MCR catalysis involves one-electron chemistry with a methyl radical intermediate that was originally proposed on the basis of computational studies^22^ and subsequently supported via *in vitro* rapid kinetic and spectroscopic experiments.^23^ The major previous mechanistic proposal involved nucleophilic chemistry with a methyl-Ni(III) intermediate similar to B_12_ chemistry.^24,25^ In the canonical version of the radical mechanism, the reaction begins with CH_3_-S-CoM coordinated to Ni(I) of coenzyme F_430_. Several crystal structures and spectroscopic experiments have revealed that HS-CoM (a substrate analog and inhibitor) is bound to the Ni(II) of F_430_ through its thiolate group.^12^ Thus, CH_3_-S-CoM was proposed to bind in a similar manner, with the thioether sulfur interacting with the catalytic Ni(I) (Figure 2C). Upon binding of HS-CoB, Ni(I) reacts with CH_3_-S-CoM, inducing homolytic cleavage to yield a transient methyl radical and a Ni(II)-S-CoM intermediate. In the next step, the methyl radical abstracts the hydrogen atom from HS-CoB to produce methane and •S-CoB, which then reacts with the F_430_-bound CoM to generate a disulfide radical anion. One-electron transfer to F_430_ then releases the heterodisulfide and regenerates Ni(I) (Figure 2C). More recently, evidence for an alternate binding mode for CH_3_-S-CoM was obtained in which the sulfonate is coordinated to the Ni of F_430_ instead of the thioether.^26^ This is an appealing scenario since it places the relevant portions of CH_3_-S-CoM and HS-CoB in close proximity to facilitate the reaction. This alternate binding scheme necessitates long-range electron transfer through CoM (Figure 2D).

To date, the *in vitro* enzymatic activity of MCR from any ANME has not been reported. Thus, the kinetic and mechanistic features of these enzymes remain largely unknown. In support of MCR-catalyzed methane oxidation, the well-studied methanogenic MCR from *M. marburgensis* can catalyze the methane oxidation reaction *in vitro*, but the specific activity was only ∼0.01% of the methane formation direction (11.4 nmol/min/mg vs 100 μmol/min/mg) and saturation of the enzyme was not observed up to ∼1 mM methane.^27^ However, these kinetic characteristics are still consistent with extrapolated parameters from AOM cultures (see Table S1) and other work has suggested *K*_*m*_ values for methane as high as 37 mM.^28^ Thus, although methanogenic MCR is clearly less efficient in catalyzing methane oxidation compared to methane formation, the fact that the enzyme can perform the extremely difficult anaerobic C–H activation of methane *in vitro* supports the use of a reverse methanogenesis pathway in ANME and highlights the possibility for MCR-based methane conversion applications.

In addition to MCR-catalyzed methane oxidation, more recently discovered MCR homologs—ACRs—perform the anaerobic oxidation of nonmethane short-chain alkanes, such as butane, propane, and ethane. Archaea in the *Ca*. Syntrophoarchaeum genus contain four ACR operons and can metabolize both butane and propane,^29^ while the ethane-oxidizing archaea that have been investigated so far encode a single ethyl-coenzyme M reductase (ECR) that is specific for ethane.^30,31^ Finally, other recent work revealed that ACR-containing archaea metabolize long-chain alkanes in oil-rich sediments, highlighting that MCR/ACR machinery can carry out the activation of alkane substrates ranging from C_1_–C_20_.^32,33^

Despite the vast numbers of MCRs and ACRs encoded in archaeal genomes, little is known about the catalytic properties of these diverse enzymes, and especially whether some may be tuned for methane oxidation vs methane formation. This concept of catalytic bias has been well-described in a handful of redox enzymes such as hydrogenases and, although not often experimentally demonstrated, catalytic bias is thought to be pervasive among enzymes.^34^ However, the lack of biochemical studies on diverse MCRs, especially from organisms carrying out methane oxidation, has so far prevented us from addressing this possibility in the MCR field. Table S1 summarizes kinetic information available for MCRs, which is still very limited.

An important discovery involving differences in the activities of MCRs came early from work on MCR isozymes. Some methanogens, including *M. marburgensis* and many other members of *Methanobacteriales* as well as some *Methanococcales* and *Methanomicrobiales,* contain two MCR isozymes. MCR isozyme I—the well-studied *M. marburgensis* enzyme described above—is more highly expressed during late stages of growth when methanogenic substrate (H_2_/CO_2_) availability is low. On the other hand, MCR isozyme II predominates during exponential stages of growth when methanogenic substrate availability is high.^35^ Further, *in vitro* studies revealed significant differences in catalytic properties,^36^ where isozyme II exhibits moderately higher *K*_*m*_s for both substrates as well as a 3.5-fold higher specific activity compared to the more well studied isozyme I (Table S1). It is important to note that these specific activity values may not be directly comparable since the two enzymes exhibited differences in rates of deactivation *in vitro*.^36^ Nevertheless, as we seek to develop an optimized methane oxidation catalyst, the capabilities of MCR isozyme II should be reevaluated.

## Structural Conservation and Divergence

Several methanogenic MCR structures are available in the Protein Data Bank, whereas only one ANME crystal structure has been reported so far, which is an ANME-1 MCR purified from Black Sea mats.^37^ Additionally, a crystal structure of ECR from *Ca*. Ethanoperedens thermophilum was recently reported.^38^ All these structures are the inactive F_430_-Ni(II) state and most contain HS-CoB and the demethylated HS-CoM.

MCR structures are highly conserved overall, consisting of a dimer of heterotrimers with two active sites harboring coenzyme F_430_ at the bottom of a ∼50 Å substrate channel (Figure 2B).^20^ The two active sites have been proposed to function as a two-stroke engine where binding of the substrates in one active site induces a conformational change that facilitates release of the heterodisulfide product in the other active site.^39^ A key connection between the active sites is via the lower axial Gln ligand to F_430_, where F_430_ bound to α is ligated by a Gln residue from α′, and vice versa.^12^ This Gln residue may also have a role in tuning the redox potential of the Ni center.^20^ The Gln coordination is well-conserved among methanogenic and ANME MCRs; however, ECR from *Ca*. E. thermophilum contains a methionine in this position, which may influence the reactivity of the F_430_.^14^

**Figure 3:**
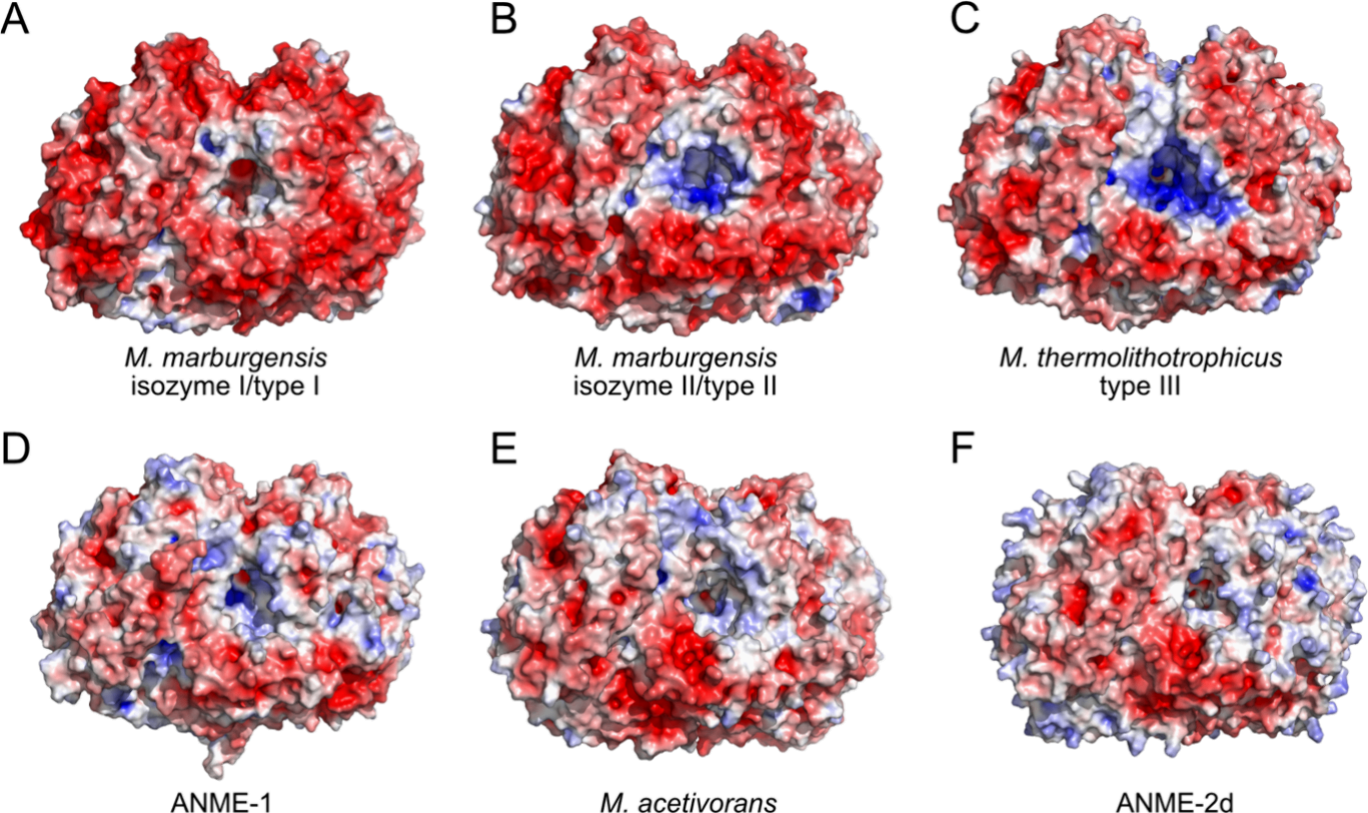
Electrostatic surface potential comparison of different MCRs. (A) *M. marburgensis* MCR I (type I, PDB 5A0Y). (B) *M. marburgensis* MCR II (type II, PDB 5A8R). (C) *Methanothermococcus thermolithotrophicus* MCR (type III, PDB 5N1Q). (D) ANME-1 MCR (PDB 3SQG). (E) *M. acetivorans* MCR.^41^ (F) AlphaFold 3 model of ANME-2d MCR.

The most noticeable overall structural difference among MCRs is the electrostatic surface potential, especially near the entrance to the substrate-binding channel. This has been previously discussed for methanogenic MCRs, where Wagner et al.^40^ defined three MCR types: type I (*Methanobacteriales*), type II (*Methanobacteriales* and *Methanococcales*), and type III (*Methanococcales*) **(**Figure 3A–C). Type III MCR has the highest number of basic residues at the entrance to the active site **(**Figure 3C), which was suggested to aid in recruiting the negatively charged substrates.^40^ Here, we compare the electrostatic surface potentials of types I–III MCRs alongside *Methanosarcina acetivorans* MCR,^41^ the ANME-1 MCR,^37^ and an AlphaFold 3^42^-generated *Ca*. M. nitroreducens MCR (ANME-2d) **(**Figure 3**D-F)**. ANME-1 MCR belongs to a distinct phylogenetic cluster while ANME-2d and *M. acetivorans* MCRs belong to the same MCR cluster.^40^ Interestingly, the electrostatic surface potentials of these three MCRs are similar, which are more neutral compared to types I–III MCRs. The differences in the electrostatic surface potentials of the various MCR types could reflect differences in folding and stability, interaction partners, and/or cellular localization.^43^ Additionally, the electrostatic characteristics of different MCRs could impact catalysis by influencing the reorganization free energy.^43^ Chadwick et al. recently offered the evolutionary idea that the original *Methanosarcinaceae* was an ANME.^17^ Thus, we suggest that the phylogenetically related MCRs with similar electrostatic characteristics (Figure 3D–F) may be optimized for catalyzing methane oxidation compared to types I–III methanogenic MCRs.

**Figure 4:**
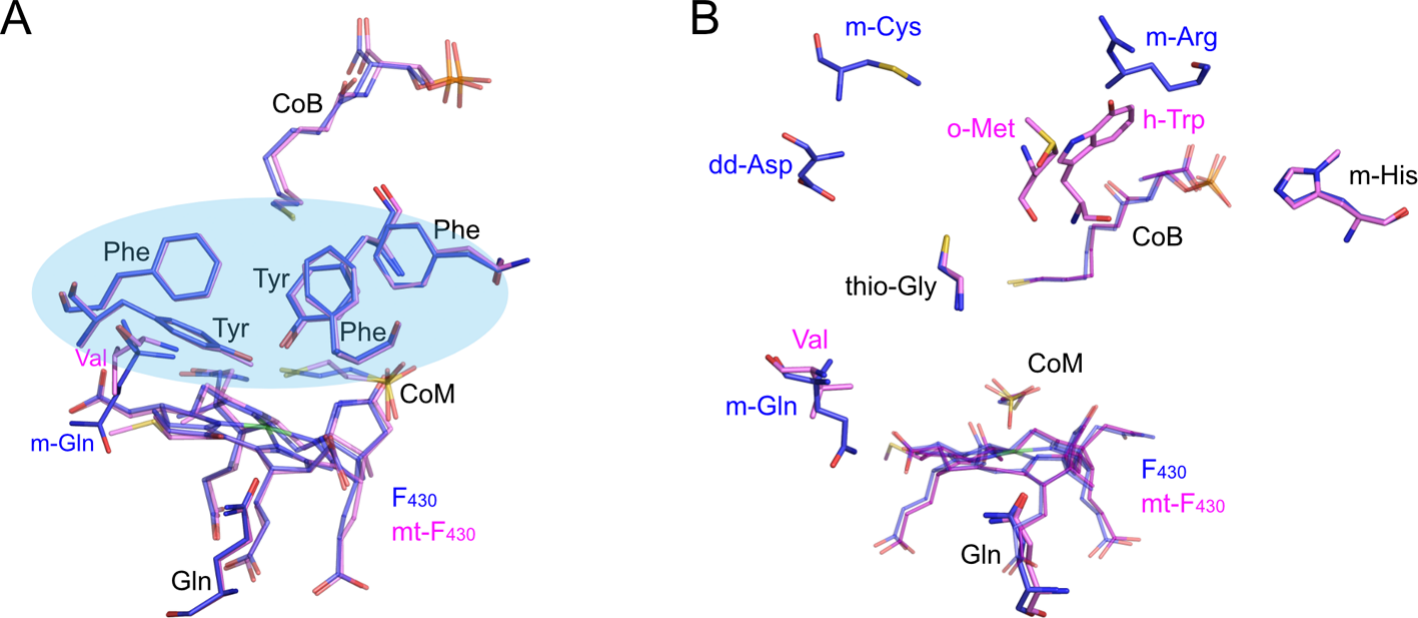
Active site views of *M. marburgensis* MCR I (blue) aligned with ANME-1 MCR (pink). (A) Conserved hydrophobic cage residues highlighted with blue oval. (B) PTM-containing amino acids. m-Cys, S-methylcysteine; dd-Asp, didehydroaspartate; m-Gln, 2-(S)-methylglutamine; thio-Gly, thioglycine; o-Met, S-oxymethioine; m-Arg, 5-(S)-methylarginine; h-Trp, 7-hydroxy-tryptophan; m-His, 1-*N*-methylhistidine. Thio-Gly and m-His modifications are present in both structures.

To investigate the role of the MCR active site electronic environment in driving the methane synthesis reaction, we recently performed electric field calculations based on molecular dynamics (MD) simulations with *M. acetivorans* MCR and ANME-1 MCR.^1^ Such fields acting on the thioether S-CH_3_ bond of CH_3_-S-CoM are thought to facilitate its homolytic cleavage. Pronounced differences in the effective electric field were observed in the two systems, which suggests that the two MCRs have differences in catalytic capabilities. Interestingly, the ANME-1 MCR active site better optimizes the electric field, indicating that ANME-1 MCR may have an enhanced catalytic efficiency compared to *M. acetivorans* MCR. Further calculations revealed that five conserved aromatic residues, comprising a hydrophobic cage surrounding CoM and the space between CoM and CoB in the MCR active site (Figure 4A), are responsible for up to half of the magnitude of the effective electric field; thus, highlighting the key role of these residues in promoting catalysis. This work was performed with the methane formation substrates, so further MD simulations and analyses need to be performed in the methane oxidation direction. Additionally, a complete quantitative confirmation of the magnitudes observed in these initial studies need to be reexamined by models accounting for more robust electrostatic properties, such as electronic polarization and charge transfer. Since ANME-1 MCR presumably operates in the methane oxidation direction *in vivo*, our results were initially surprising. However, it could be that the enzyme is an overall better catalyst in both directions, allowing it to function efficiently in the oxidation direction when methane concentrations are high.

An intriguing aspect of MCRs/ACRs is the presence of several unusual post-translational modifications (PTMs) on the McrA subunit, including thioglycine and various methylated or oxidized amino acid residues that vary depending on the organism (Figure 4B). Their distribution in different organisms, biosynthesis, and potential functions have been recently discussed^12,14^ and will not be elaborated upon here for the sake of brevity. Although the specific roles of the PTMs with respect to MCR catalysis, assembly, and/or stability remain largely unknown, they are likely involved in fine-tuning the activity of the enzyme as they are all located near the active site (Figure 4B).

## Coenzyme F_430_ Modifications

**Figure 5:**
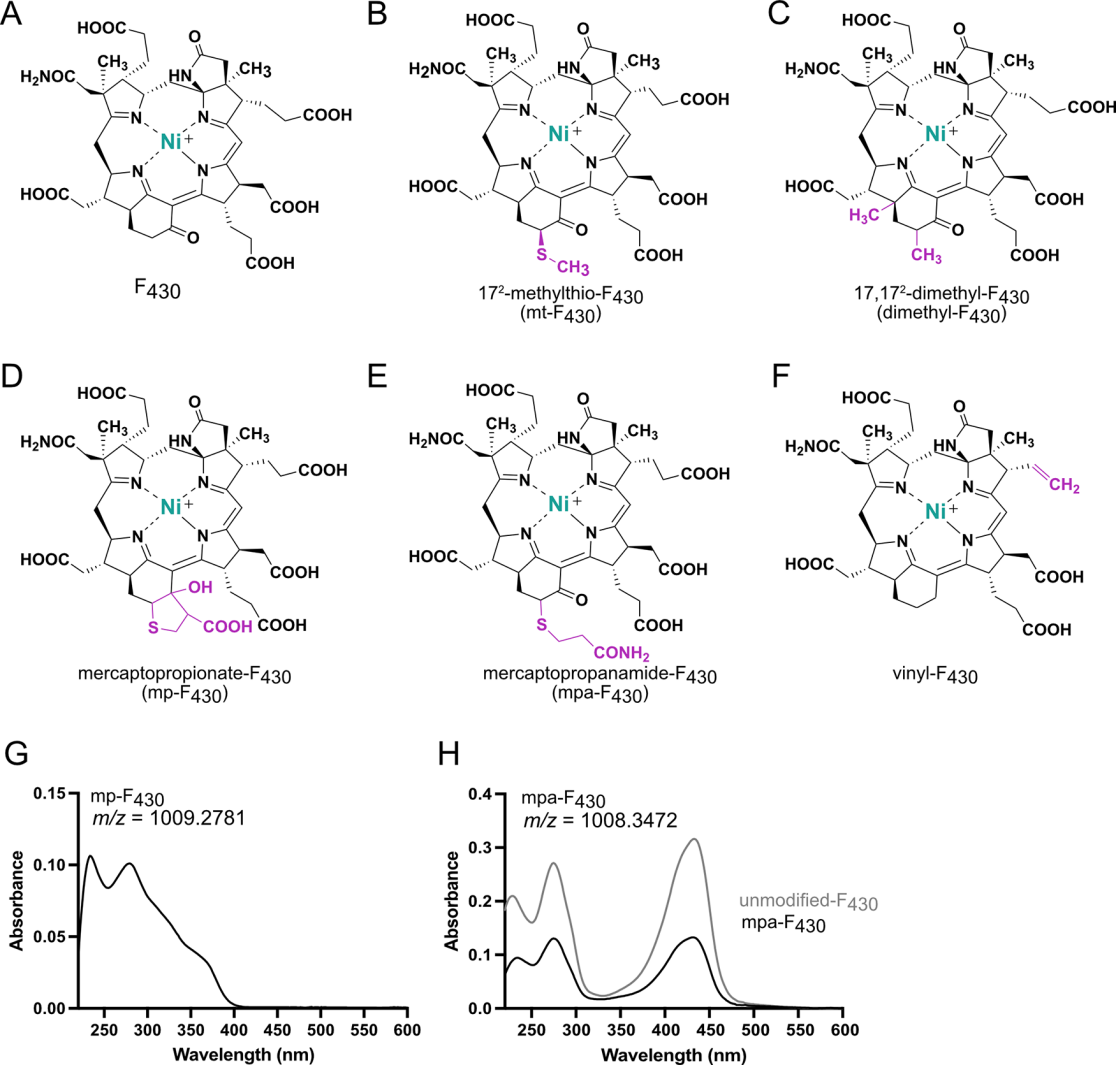
Structures of F_430_ and modified versions. (A) Canonical F_430_. (B) mt-F_430_ of ANME-1 MCR. (C) Dimethyl-F_430_ of ECR. (D–F) Tentatively proposed structures of modified F_430_s identified in some methanogens. (G) Unique absorbance spectrum of mp-F_430_. (H) Absorbance spectrum of mpa-F_430_, which is like the unmodified F_430_ (gray). mt-F_430_ and dimethyl-F_430_ also have a characteristic F_430_ absorbance spectrum.

The central catalytic component of MCRs and ACRs is coenzyme F_430_. Several modified versions of F_430_ have been discovered in a variety of different organisms. The first modified F_430_ reported was 17^2^-methylthio-F_430_ (mt-F_430_, Figure 5B), which was identified and structurally characterized from Black Sea mat samples enriched with ANME-1^44^ and later confirmed in the crystal structure of the ANME-1 MCR.^37^ The impact of this modification on MCR catalysis is unknown, but it is appealing to consider that the modification could play a role in tuning the enzyme for methane oxidation. However, it is important to note that other clades of ANME appear to utilize the canonical F_430_.^45^ More recently, a dimethyl-F_430_ was identified in the crystal structure of ECR **(**Figure 5C),^38^ which was hypothesized to maintain the structure and reactivity of the coenzyme in the expanded active site.^38^

We have identified three major F_430_ modifications in methanogens, the structures of which we currently tentatively assign as mercaptopropionate-F_430_ (mp-F_430_), the related mercaptopropanamide-F_430_ (mpa-F_430_), and vinyl-F_430_ (Figure 5D–F). Importantly, the two modified F_430_s identified in ANME-1 MCR and ECR are likely the primary physiologically relevant versions of the coenzyme since they were identified in the crystal structures of each respective enzyme. On the other hand, the canonical F_430_ is generally dominant in methanogens and work in our lab has revealed that the production of modified F_430_s in methanogens varies depending on the organism as well as the growth conditions.

The proposed mp-F_430_ was originally identified in *Methanocaldococcus jannaschii*, and the structure was assigned based upon high-resolution mass spectrometry data along with the UV–vis spectrum and some chemical characterization.^2^ The exact mass indicated the addition of a mercaptopropionate moiety, while the methyl ester derivative confirmed the presence of six carboxylic acids (one additional compared to the canonical F_430_), indicating that the mercaptopropionate group is attached as a thioether. Interestingly, the UV–vis absorbance spectrum lacked a 430 nm absorbance peak and instead displayed a broad shoulder from about 330–380 nm (Figure 5G). This is comparable to the spectrum reported for an F_430_ derivative containing a hydroxyl group instead of the ketone functionality at the 17^3^ position.^46^ This inspired our proposed cyclized structure that would result in the observed exact mass and explain the altered absorbance spectrum. More recently, we have identified a modified F_430_ in various methanogens, including *M. marburgensis* and *M. acetivorans*, that is about one mass unit less than mp-F_430_. Due to the similar masses and that we have observed both versions some methanogens, we hypothesize that these F_430_ modifications are related. The newly identified version has the same absorbance spectrum as the canonical F_430_ (Figure 5H); thus, we propose the amide-containing linear modification of mpa-F_430_ (Figure 5E). For vinyl-F_430_ (Figure 5F), there are three potential locations for an oxidative decarboxylation of a propionate side chain to produce the corresponding vinyl side chain. Notably, the vinyl modification can be compared to vinyl substituents of heme, which influence the redox properties of heme proteins.

To investigate the potential roles of F_430_ modifications on the structure and dynamics of the MCR active site, we employed MD simulations.^1^ In this work, we studied the *M. acetivorans* MCR^41^ in comparison to an ANME-1 MCR.^37^ Overall, simulations of the two MCRs with their cognate F_430_ coenzymes—*M. acetivorans* MCR with F_430_ and ANME-1 MCR with mt-F_430_ (Figure 5A and 5B)—revealed stable active site structures with the distances between the relevant portions of the cofactors remaining consistent. Val419 in the α subunits of ANME-1 MCR instead of a (methylated)-glutamine residue in other MCRs was observed to be responsible for accommodating the methylthio group at the 17^2^ position (Figure 4A).^37^ To test the impact of the methylthio group of mt-F_430_ in maintaining active site structure, we simulated ANME-1 MCR with unmodified F_430_. Indeed, we observed more flexibility and the distance between CH_3_-S-CoM and F_430_ was more variable among replicate simulations and between the two active sites, indicating that ANME-1 MCR is optimized for mt-F_430_.

**Figure 6:**
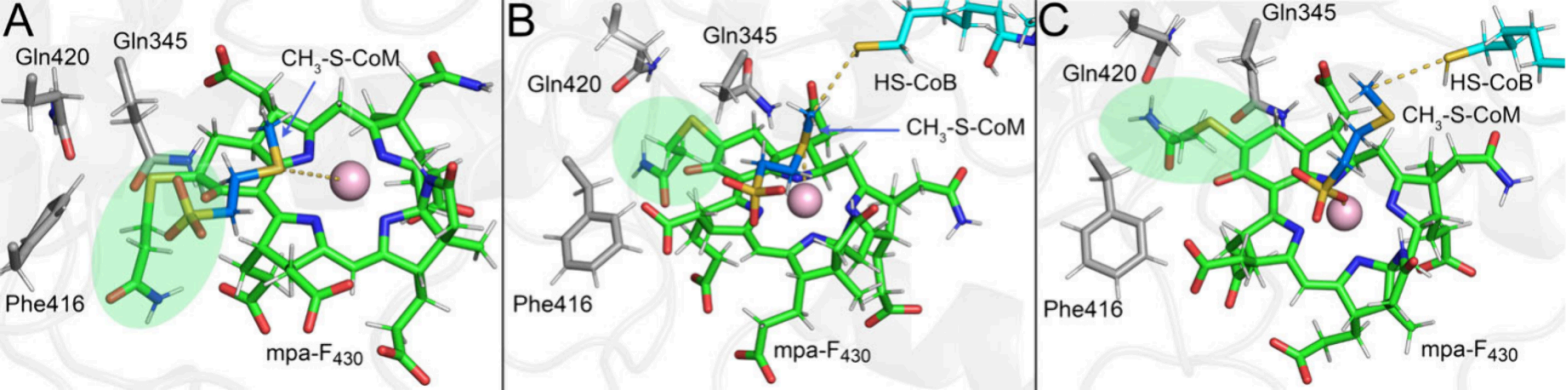
Different poses observed in molecular dynamics simulations of *M. acetivorans* MCR with mpa-F_430_. (A) Perturbed state in which the canonical active site structure and coordination between cofactors is lost. (B) A replicate in which the mercaptopropanamide modification was accommodated between Phe416 and Gln345 to maintain the canonical cofactor coordination. (C) A replicate showing the alternate CH_3_-S-CoM binding pose where the sulfonate is interacting with Ni(I). Key MCR residue are shown in gray sticks, mpa-F_430_ in green, CH_3_-S-CoM in blue, and HS-CoB in cyan. The mpa group of the modified F_430_ is highlighted with green oval. Adapted from Polêto et al.^1^ Copyright 2024 American Chemical Society.

We additionally performed MD simulations with *M. acetivorans* MCR in the presence of mpa-F_430_ as well as mt-F_430_.^1^ For the simulations with mpa-F_430_, which has not yet been captured in a crystal structure, we applied a transformation protocol^47^ in which the unmodified F_430_ was progressively converted into mpa-F_430_ in a series of independent MD simulations. This allowed the protein to gradually adjust to the coenzyme modification, thus creating a reasonable starting conformation for further unbiased MD simulations. In most replicates, modifications at the 17^2^ positions resulted in the expected steric clash with Gln420 of *M. acetivorans* MCR, which perturbed the canonical coordination among cofactors (Figure 6A). However, in one replicate, the mercaptopropanamide group displaced Gln420 and the active site reorganized to accommodate the modified F_430_ in a way that maintained the expected coordination between cofactors (Figure 6B). Interestingly, in another replicate, the mercaptopropanamide group adopted an extended conformation that disrupted the canonical thioether sulfur-Ni(I) coordination between CH_3_-S-CoM and mpa-F_430_. Instead, CH_3_-S-CoM interacted with mpa-F_430_ through its sulfonate group (Figure 6C), similar to the pose recently proposed by Patwardhan et al. (Figure 2D).^26^ This pose was never observed in our simulations with the canonical F_430_. Taken together, the active site of *M. acetivorans* MCR as captured in the crystal structure appears optimized for the canonical F_430_, although a 17^2^ modified F_430_ can be accommodated through active site reorganization. The modifications could potentially play a role in optimizing the positions of substrates/products to facilitate catalysis or product release, and/or they may impact the redox properties of the coenzyme. Indeed, recent computational calculations of the F_430_ Ni(II)/Ni(I) redox potential compared to its biosynthetic precursors showed that the values varied substantially, with the redox potential becoming steadily more positive going from earlier precursors in the biosynthesis (i.e., Ni-sirohydrochlorin *E*° = −1.77 V) to the final F_430_ (*E*° = −0.53 V).^48^ Therefore, additional modifications to F_430_ likely alter the redox properties and reactivity of the coenzyme. In addition to understanding their functions, it will be important to uncover the enzymes involved in the biosynthesis of all modified F_430_s described so far (Figure 5).

## Considerations for MCR-Based Methane Activation to Produce Commodity Chemicals

Due to the complexity of MCR in terms of its production (three subunits assembled with coenzyme F_430_ and unique PTMs) and its activation (F_430_-Ni(I)), MCR-based methane bioconversion platforms in the near term will likely be limited to methanogens or ANME as opposed to more traditional hosts for biomanufacturing. Since ANME exhibit low growth rates and low cell densities, and are not yet amenable to genetic manipulation, a methanogenic host is currently the most practical choice. Among methanogens, *M. acetivorans* has robust and well-established genetic tools including efficient CRISPR/Cas9 genome editing,^49^ and has been employed successfully in several pilot methane oxidation studies (discussed below) as well as other biotechnologically relevant metabolic engineering studies.^50^

To demonstrate the prospect of methane oxidation metabolism in a methanogen host, *M. acetivorans* expressing ANME-1 MCR was shown to perform AOM using Fe(III) as an electron acceptor.^51^ Additionally, an air-adapted strain of *M. acetivorans* expressing ANME-1 MCR was engineered for l-lactate production^52^ as well as for producing electricity from methane in a microbial fuel cell containing other engineered microbes.^53^ Despite the importance of these studies toward the goal of activating methane for conversion to useful products, it is still unclear whether the ANME MCR significantly facilitated methane oxidation since experiments were not reported to compare an overexpressed methanogenic MCR. Notably, other work has demonstrated that wild-type *M. acetivorans* can perform AOM using Fe(III) as an electron acceptor.^54,55^ Additionally, this methanogen is capable of growing without methane production via extracellular electron transfer to an artificial electron acceptor,^56^ which further highlights its metabolic versatility.

**Figure 7:**
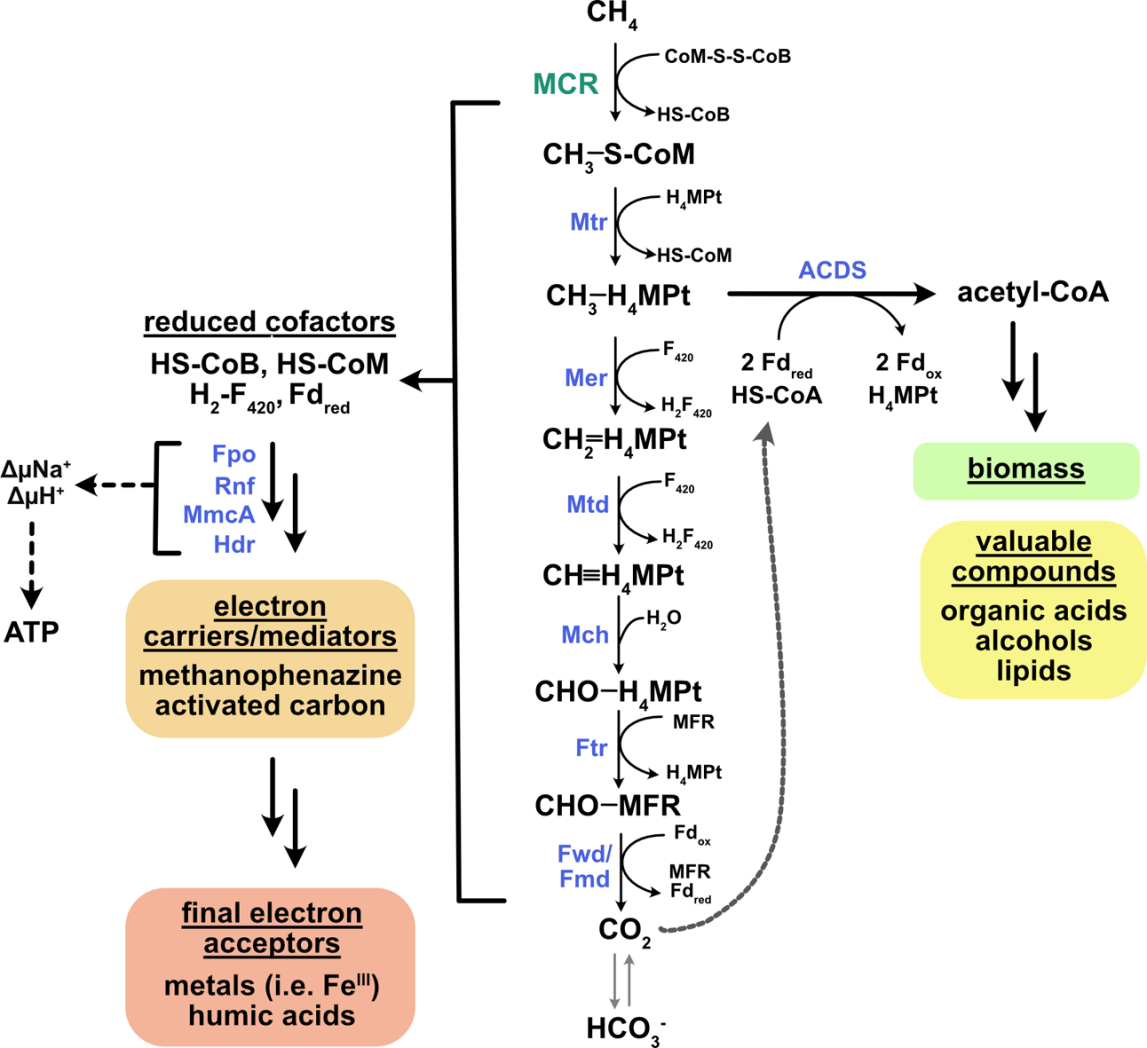
Schematic of carbon and electron flow during anaerobic methane oxidation. Abbreviations for cofactors and enzymes not in text: H_4_MPT, tetrahydromethanopterin; F_420_, coenzyme F_420_; MFR, methanofuran; Fd, ferredoxin; Mtr, methyl-H_4_MPT:coenzyme M methyltransferase; Mer, F_420_-dependent methylene-H_4_MPT reductase; Mtd, F_420_-dependent methylene-H_4_MPT dehydrogenase; Mch, methenyl-H_4_MPT cyclohydrolase; Ftr, formylmethanofuran-H_4_MPT formyltransferase; Fwd/Fmd, formylmethanofuran dehydrogenase; Rnf, ferredoxin:NAD oxidoreductase; Fpo, F_420_H_2_:methanophenazine oxidoreductase; Hdr, heterodisulfide reductase.

In a *M. acetivorans*-based methane oxidation platform (Figure 7), methane is oxidized by MCR to CH_3_-S-CoM, which continues through a reverse methanogenesis pathway to produce CO_2_ and a series of reduced cofactors. The key carbon fixation machinery entails the corrinoid iron–sulfur protein (CFeSP) and carbon monoxide dehydrogenase/acetyl-coenzyme A synthase (CODH/ACS). These three enzymes form a large protein complex in many organisms termed acetyl-coA decarbonylase/synthase complex (ACDS). CODH reduces CO_2_ to CO using two electrons from ferredoxin. This carbonyl group is then transferred to ACS for acetyl-coA synthesis along with a methyl group from CFeSP via methyl-tetrahydromethanopterin (Figure 7). Once synthesized, acetyl-CoA serves as the building block for biomolecules necessary for growth as well as for various biotechnologically useful molecules and precursors, such as acetate, lipids, and alcohols (Figure 7). The reduced cofactors generated during methane oxidation are reoxidized by donating electrons to extracellular electron acceptors such as Fe(III) or humic acids (Figure 7). Proteins involved in cofactor oxidation (Rnf, Fpo) are the energy-conserving sites. The major membrane-bound electron transfer molecule in *M. acetivorans* is methanophenazine and the key protein facilitating extracellular electron exchange in *M. acetivorans* is the multiheme c-type cytochrome, MmcA.^56,57^

In addition to methane oxidation applications with *M. acetivorans*, *Ca*. M. nitroreducens is being established as a potentially biotechnologically useful ANME. For example, modified growth conditions can promote the production of useful compounds from methane, such as acetate.^58^ The electrons gathered from these MCR-based methane oxidation processes could also be employed in bioelectrochemical technologies.^16^

## Applications of Carbon Materials to Enhance AOM

Carbon materials such as activated carbon and biochar are known to facilitate extracellular electron transfer in *Methanosarcina* cocultures^59^ and these materials also enhance methane oxidation in AOM cultures.^60,61^ Further, the potential importance of carbon materials in AOM was highlighted in our untargeted investigation of ANME biochemistry. We were intrigued by an abundant black material of unknown origin in two AOM enrichment cultures: thermophilic ANME-1a with HotSeep-1 SRB and ANME-2a/c with Seep-SRB.^3^ After removing the organic biomass components, analysis of the black material revealed three major components: magnesium phosphate (phase bobierrite), pyrite (FeS_2_), and black carbon. The latter material was observed as isolated rounded structures comprised of 90 atom % carbon. Further analysis of the carbon material by Raman spectroscopy revealed characteristic peaks corresponding to the D (disorder/defect) and G (graphite) bands, which are used to define and compare structures of carbonaceous materials. These bands exhibited nearly equal intensities in the material isolated from AOM cultures, and are consistent with the Raman spectra for amorphous carbon and pyrocarbons (activated carbon).^62^ Isotope tracing studies revealed that the amorphous carbon is only produced when methane is provided as an energy source, and is derived from dissolved inorganic carbon (DIC) and the methane-derived DIC, consistent with the origin of other biomolecules in ANME. Thus, these results demonstrate that the amorphous carbon is biochemically produced. We also identified a carbon material with similar characteristics in cultures of pure methanogens including *M. jannaschii* and *M. maripaludis*. This discovery of amorphous carbon production by ANME and methanogens is remarkable since these materials were previously only associated with abiotic production under extreme conditions (i.e., high temperature and pressure). The function of this biogenically produced carbon material is currently unclear, but it may facilitate extracellular electron transfer analogous to activated carbon and biochar (Figure 7). Redox-active functional groups associated with the carbon could act as intermediate electron donors and acceptors and/or the carbon material could provide a conductive surface for the microbes to adhere in biofilm communities to enhance redox reactions.^61,63,64^

## Concluding Remarks

MCR holds exciting potential as a methane oxidation catalyst to produce liquid fuels and other value-added chemicals. Although thermodynamics can explain the ability of MCR to operate in the direction of methane oxidation when methane concentration is high, it is apparent from the current limited data that different MCRs have different catalytic capabilities (Table S1). Thus, as we seek to develop MCR-based methane oxidation platforms, it will be important to know which enzymes may have enhanced activities in the methane oxidation direction as well as to uncover the molecular underpinnings of these differences. This would facilitate the design of optimized recombinant MCRs as well as biomimetic catalysts for methane activation applications. A major bottleneck in this area lies in the inability to purify active forms of recombinant MCRs from heterologous hosts. Notably, the recent development of genetic tools for *Methanothermobacter thermoautotrophicus* Δ*H*^65^ may open the door to obtaining active recombinant MCRs. Additionally, future work should focus on a comprehensive analysis of the *in vivo* methane oxidation abilities of different ANME MCRs compared to methanogenic MCRs overexpressed in an established AOM host, such as *M. acetivorans*. These experimental studies can be further complemented by computational analyses of diverse MCRs to assess potential differences in catalytic efficiency in the methane formation vs methane oxidation direction. Finally, extensive metabolic engineering and optimization of growth conditions will be necessary to modulate the fine balance between energy conservation, growth, and production of biotechnologically useful compounds in methanogens and/or ANME.

## Supporting Information Available

Summary of kinetic parameters of different MCRs (PDF)
