# Supplementary material for: Toward the Use of Methyl-Coenzyme M Reductase for Methane Bioconversion Applications
Source: Acc Chem Res. 2024 Aug 27;57(18):2746–57. doi: 10.1021/acs.accounts.4c00413 (PMC11411713; doi:10.1021/acs.accounts.4c00413)
Supplement: Supplementary file 1 — ar4c00413_si_001.pdf [file ar4c00413_si_001.pdf]

Supporting Information for:

Toward the Use of Methyl-Coenzyme M Reductase for Methane Bioconversion Applications

Thuc-Anh Dinh and Kylie D. Allen*
Department of Biochemistry, Virginia Tech, Blacksburg, VA 24061

*Corresponding author: kdallen@vt.edu, 540-231-5040

This PDF file includes:

Supplementary table 1

Supplementary references

Table S1. Representative kinetic parameters of different MCRs

Organism	Direction	Specific activity (μmol min^{-1} mg^{-1})	K_m (mM)	Notes	References
Methanothermobacter marburgensis	methane formation	6	CH_3-SCoM 0.6-0.8<br>HS-CoB 0.1-0.3	MCR-I at 65°C, purified in MCR_{redI} state	Bonacker et al.[1]
	methane formation	21	CH_3-SCoM 1.3-1.5 mM<br>HS-CoB 0.4-0.6 mM	MCR-II at 65°C, purified in MCR_{redI} state	Bonacker et al.[1]
	methane formation	7.8	HS-CoB 0.169 ± 0.079	MCR-I at 25°C, purified in MCR_{redI} state (60-70% of prep is active)	Wongnate et al.[2]
	methane formation	70-100	Not reported	MCR-I at 65°C, purified in MCR_{oxI} state and activated to MCR_{red1} with Ti(III) citrate	Goubead et al., Duin et al., and Mahlert et al.[3-5]
	methane oxidation	0.0114	CH_4<br>>1	MCR-I at 60°C, purified in MCR_{redI} state	Scheller et al.[6]
Methanothrix soehngenii	methane formation	0.06	CH_3-SCoM 2<br>HS-CoB 0.023	Purified MCR at 37°C	Jetten et al.[7]
Methanosarcina thermophila	methane formation	0.224	CH_3SCoM 3.3<br>HS-CoB 0.06	Purified MCR at 55°C	Jablonski and Ferry[8]
Hydrate Ridge ANME-2/SRB	methane oxidation	0.07	CH_4<br>mM range (~10)	Extrapolated from methane-dependent sulfide production in	Scheller et al., Nauhaus et al. (2007), and Nauhaus et

				laboratory cultures	al. (2002) [6, 9, 10]

References

(1) Bonacker, L. G.; Baudner, S.; Morschel, E.; Bocher, R.; Thauer, R. K. Properties of the two isoenzymes of methyl-coenzyme M reductase in *Methanobacterium thermoautotrophicum*. *Eur. J. Biochem.* **1993**, *217* (2), 587-595.
(2) Wongnate, T.; Ragsdale, S. W. The reaction mechanism of methyl-coenzyme M reductase: how an enzyme enforces strict binding order. *J. Biol. Chem.* **2015**, *290* (15), 9322-9334.
(3) Goubeaud, M.; Schreiner, G.; Thauer, R. K. Purified methyl-coenzyme-M reductase is activated when the enzyme-bound coenzyme F430 is reduced to the nickel(I) oxidation state by titanium(III) citrate. *Eur. J. Biochem.* **1997**, *243* (1-2), 110-114.
(4) Duin, E. C.; Prakash, D.; Brungess, C. Methyl-coenzyme M reductase from *Methanothermobacter marburgensis*. *Methods Enzymol.* **2011**, *494*, 159-187.
(5) Mahlert, F.; Bauer, C.; Jaun, B.; Thauer, R. K.; Duin, E. C. The nickel enzyme methyl-coenzyme M reductase from methanogenic archaea: *In vitro* induction of the nickel-based MCR-ox EPR signals from MCR-red2. *J. Biol. Inorg. Chem.* **2002**, *7* (4-5), 500-513.
(6) Scheller, S.; Goenrich, M.; Boecher, R.; Thauer, R. K.; Jaun, B. The key nickel enzyme of methanogenesis catalyses the anaerobic oxidation of methane. *Nature* **2010**, *465* (7298), 606-608.
(7) Jetten, M. S.; Stams, A.; Zehnder, A. J. B. Purification and some properties of the methyl-CoM reductase of *Methanothrix soehngenii*. *FEMS Microbiology Lettters* **1990**, *66*, 183-186.
(8) Jablonski, P. E.; Ferry, J. G. Purification and properties of methyl-coenzyme M methylreductase from acetate-grown *Methanosarcina thermophila*. *J. Bacteriol.* **1991**, *173* (8), 2481-2487.
(9) Nauhaus, K.; Albrecht, M.; Elvert, M.; Boetius, A.; Widdel, F. *In vitro* cell growth of marine archaeal-bacterial consortia during anaerobic oxidation of methane with sulfate. *Environ. Microbiol.* **2007**, *9* (1), 187-196.
(10) Nauhaus, K.; Boetius, A.; Kruger, M.; Widdel, F. *In vitro* demonstration of anaerobic oxidation of methane coupled to sulphate reduction in sediment from a marine gas hydrate area. *Environ. Microbiol.* **2002**, *4* (5), 296-305.
